# Comparative proteome analysis of Tumor necrosis factor α-stimulated human Vascular Smooth Muscle Cells in response to melittin

**DOI:** 10.1186/1477-5956-11-20

**Published:** 2013-05-07

**Authors:** Hyun-Ji Cho, Jeong-Han Kang, Kwan-Kyu Park, Jung-Yoon Choe, Yoon-Yub Park, Yong-Suk Moon, Il-Kyung Chung, Hyeun-Wook Chang, Cheorl-Ho Kim, Yung Hyun Choi, Wun-Jae Kim, Sung-Kwon Moon, Young-Chae Chang

**Affiliations:** 1 Research Institute of Biomedical Engineering and Department of Medicine, Catholic University of Daegu School of Medicine, Daegu, 705-718, Republic of Korea; 2 Laboratory of Cell Biology, NCI, National Institutes of Health, Bethesda, MD, 20892, USA; 3 Department of Biotechnology, Catholic University of Daegu, Gyeongsan, 712-702, Republic of Korea; 4 College of Pharmacy, Yeungnam University, Gyeongsan, 712-749, Republic of Korea; 5 Department of Biological Science, Sungkyunkwan University, Sungkyunkwan, Kyunggi-Do, 440-746, Republic of Korea; 6 Department of Biochemistry, College of Oriental Medicine, Dongeui University, Busan, 614-052, Republic of Korea; 7 Personalized Tumor Engineering Research Center, Department of Urology, Chungbuk National University, Chungbuk, Cheongju, 361-763, Republic of Korea; 8 Department of Food Science and Technology, Chung-Ang University, Ansung, 456-756, Republic of Korea

## Abstract

**Background:**

Bee venom has been used to relieve pain and to treat inflammatory diseases, including rheumatoid arthritis, in humans. To better understand the mechanisms of the anti-inflammatory and anti-atherosclerosis effect of bee venom, gel electrophoresis and mass spectrometry were used to identify proteins whose expression was altered in human Vascular Smooth Muscle Cells (hVSMCs) stimulated by tumor necrosis factor alpha after 12 h in the presence of melittin.

**Results:**

To obtain valuable insights into the anti-inflammatory and anti-atherosclerosis mechanisms of melittin, two-dimensional (2-D) gel electrophoresis and MALDI-TOF/TOF were used. The proteome study, we showed 33 significant proteins that were differentially expressed in the cells treated with tumor necrosis factor alpha and melittin. Thirteen proteins were significantly increased in the cells treated with tumor necrosis factor alpha, and those proteins were reduced in the cells treated with melittin. Five of the proteins that showed increased expression in the cells treated with tumor necrosis factor alpha are involved in cell migration, including calreticulin, an essential factor of development that plays a role in transcription regulation. The proteins involved in cell migration were reduced in the melittin treated cells. The observed changes in the expression of GRP75, prohibitin, and a select group of other proteins were validated with reverse transcribed-PCR. It was confirmed that the observed change in the protein levels reflected a change in the genes level. In addition, the phosphorylation of EGFR and ERK was validated by analyzing the protein pathway.

**Conclusion:**

Taken together, these data established that the expression of some proteins was significantly changed by melittin treatment in tumor necrosis factor alpha stimulated the cells and provided insights into the mechanism of the melittin function for its potential use as an anti-inflammatory agent.

## Background

The migration and proliferation of human Vascular Smooth Muscle Cells (hVSMCs) are the major causes of the development of advanced lesions in atherosclerosis [1]. The migration and proliferation of hVSMCs is caused by pathological phenomena such as the accumulation of inflammatory cells and the release of pro-inflammatory cytokines [1,2]. Pro-inflammatory cytokines such as the tumor necrosis factor (TNF)-α have various acts that mediate inflammation, and atherogenesis. Especially, TNF-α is a cytokine that is involved in systemic inflammation. Thus, the primary role of TNF-α is the regulation of immune cells. Moreover, TNF-α can induce apoptotic cell death, and inhibit tumorigenesis and viral replication [3]. On the other hand, the dysregulation of TNF-*α* production has been implicated in several of human diseases, as well as in atherosclerosis and cancer [2]. Therefore, hVSMCs and TNF-α decisively promote atherosclerosis and inflammation.

Bee venom (BV) is known as a very complex mixture of active peptides that include melittin, phospholipase A2, apamin, adolapin, hyaluronidase, dopamine, and the protease-inhibitor. It has been used in many studies on the biological and pharmacological activities that have anti-inflammatory effects on rheumatoid arthritis [4,5]. In addition, BV affects pain release, and immune modulatory activity [6]. It has also, been reported to have induced apoptosis and suppressed the signaling pathway in leukemic cells and renal cancer [7,8]. The major compound of BV is melittin, a 26 amino acid peptide, which forms an amphipathic helix with a highly charged carboxyl terminus [9]. It comprises 52% of BV peptides [10]. Melittin reportedly has multiple effects, such as antibacterial, antivirus, and anti-inflammatory effects, in various cell types [11,13]. In addition, it has been reported to be capable of cell cycle arrest, growth inhibition, and apoptosis in various tumor cells [12,13]. However, the mechanisms of the anti-atherosclerosis and anti-inflammatory effects of melittin have not yet been fully explained. Thus, the proteomics method was used to understand the mechanisms of melittin in inflammation-induced hVSMCs. Such studies can be facilitated by comparing the obtained gels, with the 2-DE reference gels representing the typical pattern of the cells being studied under normal conditions. The association of 2-D electrophoresis with MALDI-TOF-TOF mass spectrometry and database interrogations enabled the identification of 33 proteins that were differentially expressed in the hVSMCs after melittin treatment. In particular, various proteins were implicated in the inflammation, regulation of the protein folding, oxidation reduction, and signal transduction.

## Results

### Detection of the differentially expressed proteins in the hVSMCs

To determine the changes in the protein expression after the treatment of the cells with TNF-α or melittin, we used 2-D gel electrophoresis to separate the total cell proteins from the hVSMCs. Each gel was loaded with 400 μg of protein. Approximately 1,000 individual spots were resolved in this manner. It was expected that many individual spots would contain more than one protein and this was borne out by the subsequent analysis using mass spectrometry. It was also found in a number of instances that the same protein was present in multiple spots and was most likely the product of the post-translational modifications or alternative splicing at the mRNA level. Nevertheless, an estimated 900 individual proteins at least were resolved, which provided a representative samples of the cellular proteins and allowed identification of many differentially expressed proteins.

**Figure 1:**
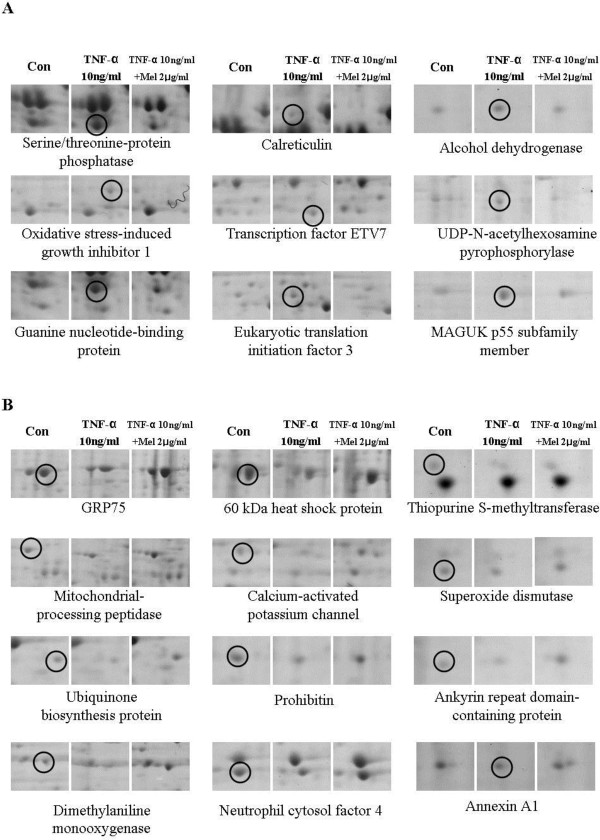
**Expression of select proteins in hVSMCs following treatment with TNF**-**α or melittin.** Whole cell extracts (400 μg of protein) were prepared from hVSMCs cultured with TNF-α or melittin. Proteins from each culture were separated in parallel by two-dimensional SDS-PAGE. Enlarged images of the spots containing proteins with increased expression in TNF-α-treated cells are shown in *panel****A***; images of spots containing proteins with decreased expression in melittin-treated cells are shown in *panel****B***.

Three gels per sample were processed simultaneously and analyzed with PDQUEST 2-D software to quantitatively compare the proteins that were recovered from the treated cultures with those from the untreated cells. Figure 1A and B show enlarged views of the gel regions that contained spots whose staining intensity was, significantly increased and decreased, respectively, after the TNF-α or melittin treatment.

### Identification of the differentially expressed proteins with MS

The protein spots from the 2-D gels were subjected to trypsin digestion and MALDI-TOF-TOF analysis. Protein was definitively identified in 33 of the 52 spots that showed significant changes, after the melittin treatment. Several proteins were identified multiple times because they were present in more than one spot. Table 1 lists the proteins with the significantly differential expressions, which had staining intensity ratios in the samples from the treated and untreated cells were ≥ 1.5 or ≤ 0.6. More than 95% of the spots had more than 10% sequence coverage and were identified with a 95% confidence level. The identities of many of the proteins were further validated by the agreement between their apparent molecular weights and the isoelectric points that were estimated from the positions of the identified proteins in the 2-D gels and their theoretical values (M_r_ < 20% or pI < 0.5).

**Table 1:** Proteins showing differential expression in hVSMCs in response to melittin

	^***a***^**AC No**	**Protein**	**Localization**	**Function**	^***b***^**Score**	**MW**	**PI**	**TNF**/**Con**	**MEL** + **TNF**/**Con**
**1**	**Q15257**	**Serine**/**threonine**-**protein phosphatase 2A regulatory subunit**	**C**	**Apoptosis**	**560**	**40**	**5.6**	**1.603**	**0.903**
**2**	**Q9Y3V2**	**RWD domain**-**containing protein 3**	**C**	**Protein binding**	**400**	**30**	**6**	**1.589**	**0.857**
**3**	**P38646**	**Stress**-**70 protein**	**M**	**Anti**-**apoptosis**	**67833**	**73**	**5.9**	**0.555**	**1.026**
**4**	**Q9UJX0**	**Oxidative stress**-**induced growth inhibitor 1**	**C**	**Ccell growth**	**112**	**60**	**7**	**3.456**	**0.47**
**5**	**O75439**	**Mitochondrial**-**processing peptidase subunit beta**	**M**	**Proteolysis**	**138**	**54**	**6.4**	**0.331**	**1.597**
**6**	**O75208**	**Ubiquinone biosynthesis protein COQ9**	**M**	**Ubiquinone biosynthesis**	**151**	**35**	**5.6**	**0.615**	**1.579**
**7**	**Q99518**	**Dimethylaniline monooxygenase**	**ER**	**Oxidoreductase**	**161**	**60**	**8.6**	**0.434**	**1.571**
**8**	**P10809**	**60 kDa heat shock protein**	**M**	**Chaperone**	**730**	**61**	**5.7**	**0.553**	**1.094**
**9**	**Q8N987**	**N**-**terminal EF**-**hand calcium**-**binding protein 1**	**C**	**Calcium ion binding**	**404**	**40**	**4.8**	**1.7**	**0.229**
**10**	**Q96AB6**	**Protein N**-**terminal asparagine amidohydrolase**	**C**	**Hydrolase**	**112**	**34**	**5.8**	**0.235**	**0.688**
**11**	**P08754**	**Guanine nucleotide**-**binding protein G**(**k**) **subunit alpha**	**C**	**Transducer**	**1704**	**40**	**5.5**	**1.631**	**0.782**
**12**	**P27797**	**Calreticulin precurso**	**ER**	**Signal transducer activity**	**183**	**48**	**4.3**	**2.225**	**0.362**
**13**	**Q9H361**	**Polyadenylate**-**binding protein 3**	**C**	**Poly**(**A**) **RNA binding**	**156**	**70**	**9.7**	**8.041**	**0.25**
**14**	**Q6IQ49**	**Uncharacterized protein C1orf55**	**Unknown**	**Unknown**	**4333**	**49**	**5.8**	**0.45**	**1.604**
**15**	**Q9NPA1**	**Calcium**-**activated potassium channel subunit beta**-**3**	**CM**	**Transport**	**920**	**31**	**6.9**	**0.589**	**2.289**
**16**	**Q9Y603**	**Transcription factor ETV7**	**N**	**Repressor**	**142**	**38**	**8.3**	**3.929**	**0.39**
**17**	**Q17RB8**	**LON peptidase N**-**terminal domain and RING finger protein 1**	**M**	**Proteolysis**	**133**	**47**	**5.6**	**0.614**	**1.261**
**18**	**P35232**	**Prohibitin**	**M**	**DNA replication**	**1501**	**29**	**5.6**	**0.567**	**1.581**
**19**	**Q9Y262**	**Eukaryotic translation initiation factor 3 subunit E**-**interacting protein**	**C**	**Initiation factor**	**1001**	**66**	**5.9**	**1.623**	**1.01**
**20**	**Q9Y512**	**Sorting and assembly machinery component 50 homolog**	**M**	**Protein binding**	**303**	**51**	**6.4**	**0.194**	**0.632**
**21**	**Q9Y3Q3**	**Transmembrane emp24 domain**-**containing protein 3 precursor**	**G**	**Transport**	**1306**	**24**	**5.4**	**0.532**	**1.024**
**22**	**Q15080**	**Neutrophil cytosol factor 4**	**C**	**Immune response**	**116**	**39**	**6.4**	**0.352**	**0.985**
**23**	**Q12792**	**Twinfilin**-**1**	**C**	**Regulation of actin phosphorylation**	**177**	**42**	**6.5**	**0.03**	**2.558**
**24**	**P31321**	**cAMP**-**dependent protein kinase type I**-**beta regulatory subunit**	**C**	**Transmembrane transport**	**219**	**43**	**5.6**	**0.504**	**1.865**
**25**	**Q15080**	**Neutrophil cytosol factor 4**	**C**	**Immune response**	**104**	**39**	**6.4**	**0.881**	**1.703**
**26**	**Q8N3R9**	**MAGUK p55 subfamily member 5**	**C**	**Tight junction assembly**	**3244**	**77**	**5.8**	**4.311**	**1.148**
**27**	**P06753**	**Tropomyosin alpha**-**3 chain**	**C**	**Actin binding**	**179**	**32**	**4.7**	**1.534**	**0.473**
**28**	**P14550**	**Alcohol dehydrogenase**	**C**	**Oxidoreductase**	**173**	**36**	**6.3**	**1.722**	**0.876**
**29**	**Q5TZF3**	**Ankyrin repeat domain**-**containing protein 45**	**C**	**Unknown**	**2335**	**31**	**4.6**	**0.739**	**2.151**
**30**	**Q16222**	**UDP**-**N**-**acetylhexosamine pyrophosphorylase**	**C**	**Transferase**	**193**	**58**	**5.9**	**4.854**	**0.304**
**31**	**P04083**	**Annexin A1**	**C**	**Anti**-**apoptosis**	**271**	**38**	**6.6**	**0.6**	**1.442**
**32**	**P51580**	**Thiopurine S**-**methyltransferase**	**C**	**Transferase**	**150**	**28**	**5.8**	**0.051**	**1.518**
**33**	**O14618**	**Copper chaperone for superoxide dismutase**	**C**	**Chaperone**	**137**	**29**	**5.3**	**0.423**	**1.27**

^*a*^ The Swiss-Prot/TrEMBL database accession number. ^*b*^ protein score greater than 100 are significant (p < 0.05). CM: Cell membrane, ER: Endoplasmic reticulum, M: Mitochondrion, N: Nucleus, C: Cytoplasm, G: Golgi apparatus, L: Lysosome, S: Secreted, MC: Microtubule.

**Figure 2:**
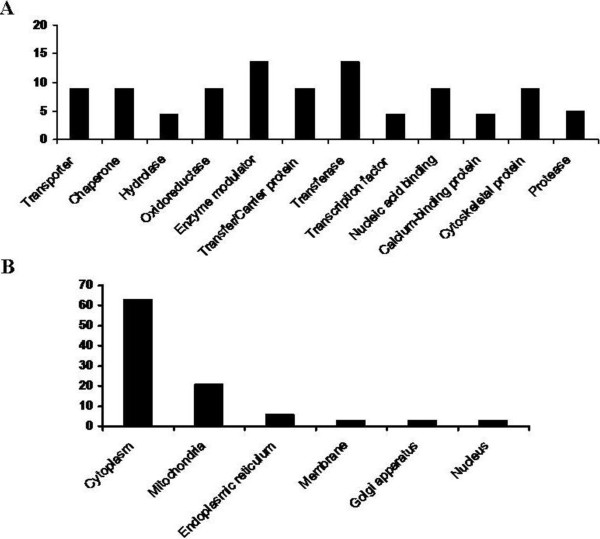
**Classification of the proteins showing differential expression.** Bar graph representing the distribution of the 33 identified proteins according to their biological function, *panel ****A***, and cellular localization, *panel ****B***. Assignments were made based on information from the NCBI (http://www.ncbi.nlm.nih.gov/PubMed) and the Swiss-Prot/TrEMBL protein knowledgebase (http://au.expasy.org/sport) websites.

The proteins were classified according to their molecular functions using the Panther Classification System (http://www.pantherdb.org) and according to their categories based cellular locations using the classifications from the Swiss-Prot/TrEMBL protein knowledge base. The distribution of the proteins into the different classes is depicted in the bar graph in Figure 2A. The functional categories with the highest representativeness were transferase (~18%), the enzyme modulator (17%), and oxidoreductase (10%). In addition, 65% of the proteins were localized in the cytoplasm (Figure 2B).

### Protein pathway analysis of the differential expression protein in the TNF-α treated cells

**Figure 3:**
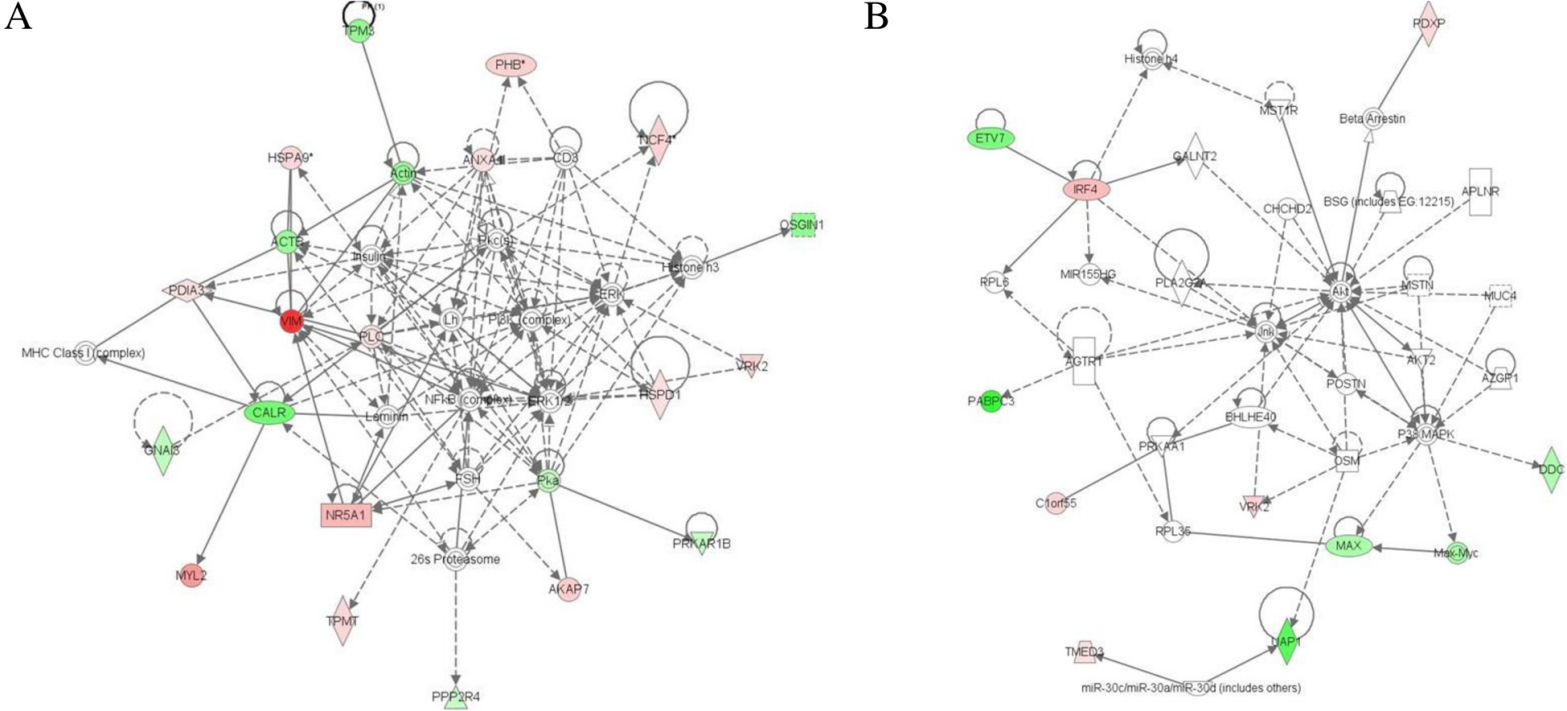
**Network pathway analysis of the differential expressed proteins melittin treatment.** Proteins are grouped as being (**A**) primarily involved in lipid metabolism, small molecule biochemistry, cellular movement, (**B**) primarily involved in developmental disorder, skeletal and muscular disorders, cancer. Proteins shaded in red showed a ≥ 1.5-fold increase in abundance in melittin treated cells. Proteins shaded in green showed a ≤ 0.6-fold decrease in abundance in melittin treated cells. The color intensity denotes the degree of abundance. Proteins were identified through the ingenuity Knowledge Base. The shapes denote the molecular class of the protein. Solid line indicates a direct molecular interaction.

To provide some insights into the cellular activities that were affected by the TNF-α or melittin treated cells, a pathway analysis was performed to place the proteins into different functional networks. As shown in Figure 3A, the 22 proteins that are involved in lipid metabolism, small molecule biochemistry and cellular movement were grouped together. In addition, the 11 proteins that are involved in developmental disorder, skeletal and muscular disorders, and cancer were grouped together (Figure 3B). Therefore, selected IPA results were validated using biochemical techniques.

### Validation of the selected proteins by with RT-PCR

**Figure 4:**
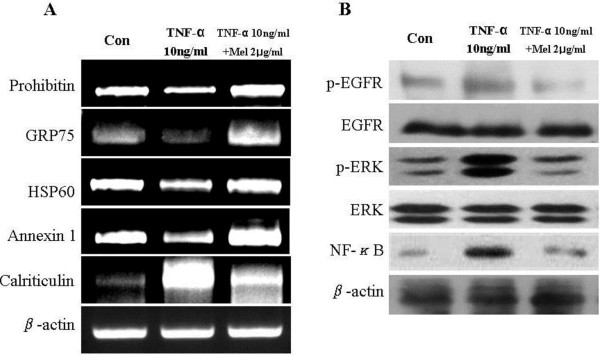
**Validation of the two**-**dimensional**-**PAGE data by quantitative RT**-**PCR and Validation of the protein pathway analysis data. *A***, expression of selected genes in hVSMCs treated with TNF-α or melittin for 12 h was determined by quantitative RT-PCR. Total RNA was isolated from hVSMCs, reverse transcribed and amplified with the specific primers indicated in under “materials and methods.” β-actin was used as the control. ***B***, Total cell lysates (25 μg) and nuclear lysates of hVSMCs treated for 12 h with either TNF-α or melittin were separated by SDS-PAGE. Proteins were blotted onto a PVDF membrane, probed with specific antibodies, and detected as described under “materials and methods.”

To confirm the changes in the protein expression after the melittin treatment, RT-PCR analysis was performed to measure the changes in the corresponding mRNAs. As shown in Figure 4A, the levels of four genes were reduced when the cells were treated with TNF-α and the levels of all these four genes increased when melittin added. Additionally, the mRNA expressions of prohibitin and HSP60 did not change when only melittin added. The expressions of GRP75 and annexin 1 increased more significantly than those of the non-stimulated cells, however, when only added with melittin (Additional file 1A). In contrast, the level of calriticulin was increased in the TNF-α treated cell and did not change when cells were treated with melittin (Additional file 1A). The mRNA measurements confirmed that the changes were observed by staining following the 2-D gel electrophoresis reflected changes in the protein levels. The analysis of the protein pathway in the melittin-treated cells also showed the phosphorylation of EGFR, and ERK and the expression of NF-κB. As shown in Figure 4B, the melittin treatment reduced the phosphorylation of EGFR, and ERK and the expression of NF-κB in nuclear. Moreover, they were reduced more significantly than the cells that were not-stimulated cells by melittin (Additional file 1B). Therefore, it is suggested that melittin blocks the level of genes and the activity of kinases.

## Discussion

BV has been widely used to relieve pain and treat inflammation in various chronic inflammatory diseases, such as rheumatoid arthritis and multiple sclerosis. A major compound of BV, melittin is the principal active component of BV and is a powerful stimulator of phospholipase A2. It also inhibits various protein kinases and is a cell membrane lytic factor. It has also shown potent anti-microbial activity and suppression of infections [14,15].

In this study, a proteomics analysis of hVSMCs treated with TNF-α or melittin was first performed to identify differentially expressed proteins that are highly affected by melittin in TNF-α stimulated cells. This technique revealed 52 differentially expressed proteins and identified 33 significantly differentially expressed proteins whose expressions had consistently different patterns after TNF-α or melittin treatment. Many of the identified proteins were Stress-70 protein and Annexin A1, which are involved in anti-apoptosis.

Prohibitin 1 (PHB) is multifunctional protein that is localized in cells and mitochondrial membranes as well as in the nucleus [16,17]. It is implicated in cellular processes such as the mitochondrial function and protein folding [18], transcription regulation [19] and proliferation control and suppression of oncogenesis [20,21]. Expression of PHB decreases during ulcerative colitis and Crohn’s disease, two forms of inflammatory bowel disease [22,23]. In this study, prohibitin expression was down-regulated in TNF-α treated cells and up- regulated in melittin treated cells.

Annexins have been diversely implicated in cell differentiation and proliferation, extracellular processes such as coagulation, and membrane fusion events such as endocytosis and exocytosis [24]. In addition, annexins are intracellular molecules that are implicated in the down-regulation of inflammation. Annexin-1 was identified recently as a secreted molecule and suggested as a potent inhibitor of inflammation both in vitro, and in vivo. Studies have indicated that annexin-1 is secreted, and subsequent reports have shown that secreted annexin-1 participates in additional anti-inflammatory effects such as detachment of neutrophils from the vascular endothelium [25,26]. In this study, annexin-1 expression was restored in melittin-treated hVSMCs and melittin was suggested as having anti-inflammatory effects.

Calreticulin binds unfolded glycosylated proteins in the ER and is implicated in many cellular functions such as lectin-like chaperoning, Ca2^+^ storage and signaling, regulation of gene expression, cell adhesion, wound healing and auto-immunity. Its over-expression on the surface of lung fibroblasts has been reported in response to cytomegalovirus infection [27,28]. Calreticulin was up-regulated by TNF-α and down-regulated in the melittin-treated hVSMCs.

## Conclusion

This is the first report of the proteomic analysis of the effects of melittin treatment on cultured hVSMCs. In this study, the anti-inflammatory mechanism of melittin on the inflammatory process was discovered. And several target molecular of inflammation and proliferation such as prohibitin, annexin-1 and calreticulin were identified. In addition, we found that two major protein pathways using bioinformatics method. The protein pathway analysis showed that NF-κB, and EGFR are the main molecular of inflammation in hVSMCs treated with TNF-α or melittin. It was consistent with other study. Jijon HB et al. reported that TNF-α induced ERK and EGFR activation [29] and Andrianifahanana M et al. reported that TGF-β induced EGFR activation [30]. According these reports, we suggest that TNF-α induced ERK/NF-kb and EGFR. We believe that this experiment would improve understanding of the anti-inflammatory effects of melittin on inflammatory disease, and atherosclerosis.

## Methods

### Cell culture and biological reagents

Human Vascular Smooth Muscle Cells (hVSMCs) were obtained from the American Type Culture Collection (Manassas, VA, USA). Cells were cultured in RPMI medium (Invitrogen, Gland island, USA) containing 10% fetal bovine serum. The cells were maintained at 37°C. The hVSMCs were grown on culture plates to 60%-70% confluence in complete medium containing 10% FBS for 12 hours, and then changed to serum-free medium after washing twice with medium. Then, the cells were incubated with TNF-α or melittin at various concentrations.

### Protein extraction and Two-dimensional gel electrophoresis

hVSMCs were washed three times with ice-cold PBS. Cells were lysed with a buffer containing 5 mM EDTA, 9.5 M urea, 4% (v/v) CHAPS, 65 mM DTT and protease inhibitors (Complete kit, Roche Diagnostics, Germany) for 1 h at 24°C. Cellular debris was removed by centrifugation for 15 min at 20000 × *g* at 4°C. Protein samples were stored at −70°C. Protein concentrations were quantified using a commercial Bradford Kit (DC reagent kit, Bio-Rad).

2-D electrophoresis was performed using an established procedure [31]. Whole cell lysate (400 μg) was added to immobilized pH 3–10 linear gradient strips (ReadyStrip IPG strip, Bio-Rad). After finished IEF, the IPG strips were incubated in equilibration buffer containing 37.5 mM Tris–HCl (pH 8.8), 6 M urea, 2% (w/vol) SDS, 30% (v/v) glycerol and 2% (w/vol) DTT or 2.5% (w/vol) iodoacetamide for 30 min. The equilibrated IPG strips were transferred onto 12% Duracryl gels (180 × 160 × 1.5 mm) for SDS–PAGE. We stained gels with sensitive colloidal coomassie G-250 according to Neuhoff et al. [32]. To check the reproducibility of the data, three independent experiments were performed on each cell lysate. For the differential analysis, statistical significance was estimated with Student’s *t*-test. Values of *p* < 0.05 were considered significant.

### Protein identification

Protein identification was performed as described previously study [33]. In brief, proteins were in-gel digested with trypsin and extracted from coomassie stained 2-D gel pieces in according to standard procedures. After in-gel digested, the peptides were extracted twice with 0.1% TFA in 50% acetonitrile. Extracts were pooled and lyophilized. The resulting lyophilized tryptic peptides were concentrated and desalted by passing them through C18ZipTip (Millopore, Billerica, MA, USA) following standard procedures. MS analysis was conducted with a MALDI-TOF-TOF mass spectrometer 4700 Proteomics Analyzer (Applied Biosystems, Framingham, MA, USA). Data were analyzed using GPS Explorer software (Applied Biosystem) and MASCOT software (Matrix Science, London, UK). NCBInr and human were selected as the database and taxonomy, respectively. Identification was assigned to a protein spot feature if the protein score was calculated to be greater than 50, correlating to a confidence interval of 99%.

### Protein pathway analysis

After protein identification, the accession numbers and fold changes of the differentially expressed proteins were tabulated in Microsoft Excel and imported into IPA (Ingenuity System, Montain View, CA, USA). IPA is a software application that enables to identify the biological mechanisms, pathways and functions matching a particular dataset of proteins. IPA is based on a database obtained by abstracting and interconnecting a large fraction of the biomedical literature according to a very strict algorithm. This database integrates protein functions, cellular localization, small molecules and disease inter-relationships. The networks are displayed graphically as nodes, representing individual proteins and edges representing the biological relation between nodes. Using IPA, Canonical pathway analysis utilizes well characterized metabolic and cell signaling pathways which are generated prior to data input and on which identified proteins are overlaid.

### Western blot analysis

Whole cell and nuclear lysates were prepared as previously described [31]. Cells were lysed with RIPA buffer. (50 mM Tris, 150 mM NaCl, 1 mM EDTA, 1 mM DTT, 0.5% [v/v] NP 40 10 mM NaF and proteases inhibitors). The cells were disrupted and proteins were extracted at 4°C for 30 min. The proteins were electro transferred to PVDF membranes (Invitrogen). Detection of specific proteins was carried out with an enhanced chemiluminescence western blotting kit following the manufacturer’s (Pierce) instructions. Antibodies specific for p-ERK, ERK, EGFR, p-EGFR, NF-κB and β-actin were purchased from Santa Cruz Biotechnology (Santa Cruz, CA).

### Reverse transcribed–PCR-analysis

After treatment of cells with TNF-α or melittin, Total RNA was isolated from each preparation using the Trizol reagent (Invitrogen, Carlsbad, CA). Reverse transcription was carried out using a commercial kit (Superscript II RNase H-reverse transcriptase, Invitrogen) and total RNA (1 μg) from hVSMCs, according to the manufacturer's protocol. Gene expression was analyzed using specific primers. Amplified products were resolved by 1.0% (w/v) agarose gel electrophoresis and visualized by staining with ethidium bromide. We quantified the actual mRNA level of each gene by using Eagle Sight densitometry software (Version 3.21; Stratagene, La Jolla, CA).

## Competing interests

The authors declare that they have no competing interests.

## Authors’ contributions

HJC and JHK performed proteomic analysis including its design, coordination, analysis of the data, and drafted the manuscript. All authors read and approved the final manuscript.

## Supplementary Material

Additional file 1 **Validation of the two-dimensional-PAGE data by quantitative RT-PCR and validation of protein pathway analysis data. *A***, expression of selected genes in hVSMCs respectively treated with TNF-α and melittin for 12 h was determined by quantitative RT-PCR. Total RNA was isolated from hVSMCs, reverse transcribed and amplified with the specific primers indicated in under “Materials and methods.” β-actin was used as the control. ***B***, Total cell lysates (25 μg) and nuclear lysates (40 μg) of hVSMCs respectively treated for 12 h with TNF-α and melittin were separated by SDS-PAGE. Proteins were blotted onto a PVDF membrane, probed with specific antibodies, and detected as described under “materials and methods.” Click here for file
