# Supplementary material for: Comparative proteome analysis of Tumor necrosis factor α-stimulated human Vascular Smooth Muscle Cells in response to melittin
Source: Proteome Sci. 2013 May 7;11:20. doi: 10.1186/1477-5956-11-20 (PMC3655938; doi:10.1186/1477-5956-11-20)

## SUPPLEMENT DATA 1. **Validation of the two-dimensional-PAGE data by quantitative RT-PCR and validation of protein pathway analysis** **data.** *A*, expression of selected genes in hVSMCs respectively treated with TNF-α and melittin for 12 h was determined by quantitative RT-PCR. Total RNA was isolated from hVSMCs, reverse transcribed and amplified with the specific primers indicated in under “Materials and methods.” β-actin was used as the control. *B*, Total cell lysates (25 μg) and nuclear lysates (40 μg) of hVSMCs respectively treated for 12 h with TNF-α and melittin were separated by SDS-PAGE. Proteins were blotted onto a PVDF membrane, probed with specific antibodies, and detected as described under “materials and methods.”

##
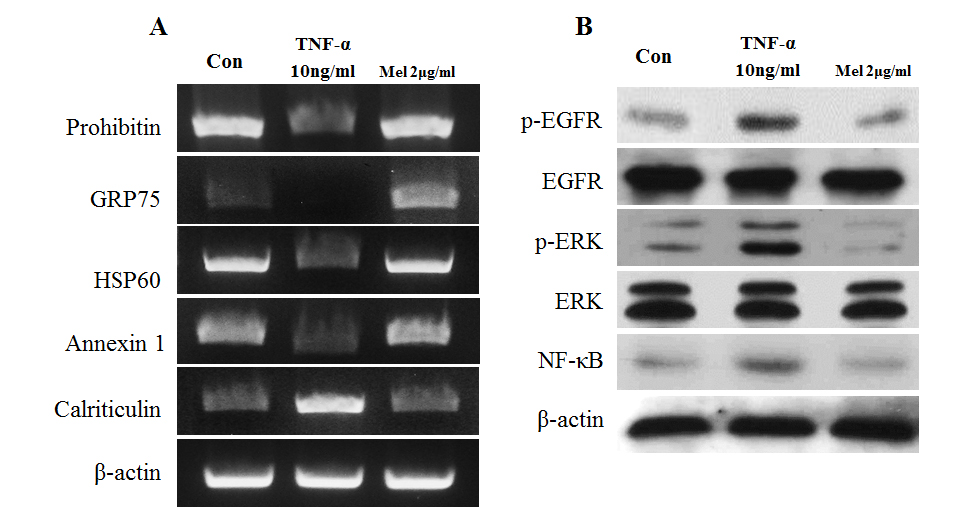

Supplement: Additional file 1 — Validation of the two-dimensional-PAGE data by quantitative RT-PCR and validation of protein pathway analysis data. A, expression of selected genes in hVSMCs respectively treated with TNF-α and melittin for 12 h was determined by quantitative RT-PCR. Total RNA was isolated from hVSMCs, reverse transcribed and amplified with the specific primers indicated in under “Materials and methods.” β-actin was used as the control. B, Total cell lysates (25 μg) and nuclear lysates (40 μg) of hVSMCs respectively treated for 12 h with TNF-α and melittin were separated by SDS-PAGE. Proteins were blotted onto a PVDF membrane, probed with specific antibodies, and detected as described under “materials and methods.” [file 1477-5956-11-20-S1.doc]
